# One-Step Genotyping Method in loxP-Based Conditional Knockout Mice Generated by CRISPR-Cas9 Technology

**DOI:** 10.1007/s12033-022-00500-5

**Published:** 2022-05-03

**Authors:** He Zhu, Siqian Liu, Wenxi He, Fei Sun, Yang Li, Ping Yang, Qilin Yu, Shu Zhang

**Affiliations:** 1 Department of Respiratory and Critical Care Medicine, The Center for Biomedical Research, NHC Key Laboratory of Respiratory Diseases, Tongji Hospital, Tongji Medical College, Huazhong University of Science and Technology, Wuhan, Hubei Province, China; 2 Department of Pharmacy, Tongji Hospital, Tongji Medical College, Huazhong University of Science and Technology, Hubei Province, Wuhan, China

**Keywords:** Genotyping, Tetra primer-paired PCR amplification, PCR-based protocol, loxP allele

## Abstract

With the development of CRISPR-Cas9 gene editing and in vitro fertilization (IVF) technology, we can now easily construct genetically modified mouse strains with indels, especially for loxP-based strategy. However, the general genotyping methods are time-consuming and unreliable given the loxP site is only 34 bp long. Here, based on the tetra primer-paired PCR amplification, we describe an efficient genotyping method which can simultaneously generate the internal control band, wild type (wt)-genotype band, and/or loxP-genotype band through one single PCR amplification. It is easy to interpret the mouse genotypes from the pattern of the bands. Further, the results could also help to exclude the possibility of minor cross-contamination, since the ratio between the bands’ quantity in wt/wt, wt/loxP, and loxP/loxP mice are relatively constant, which makes the genotyping more reliable when it is performed in a large amount.

**Supplementary Information:**

The online version contains supplementary material available at 10.1007/s12033-022-00500-5.

## Introduction

Cre-loxP system is a site- and cell type-specific recombination system that has been widely applied for spatiotemporal gene manipulation. LoxP (locus of X-over of P1) is a 34 base pair (bp) long DNA element consisting of two 13 bp inverted repeats separated by an 8 bp nonpalindromic (asymmetric) sequence, which dictates the orientation of the overall loxP site [1]. With the development of CRISPR-Cas9 gene editing system, a cost-effective methodology has been established to construct transgenic embryos, rapidly and accurately [2]. However, as the advancing genome-editing technologies facilitate the construction of transgenic animal models and human cell models, genotyping technologies are lagging behind and becoming a bottleneck.

So far, the gold standard for detecting modified genes is sequencing PCR products of target gene regions using Sanger method, which is costly and labor intensive [3]. Meanwhile, nuclease-based methods such as T7 Endonuclease I (T7E1) [4] and Surveyor nuclease [5] often underestimate indel frequencies [6], and are unreliable when the indel frequency is over 30% or < 3% [7]. Recently, both PAGE and qPCR-based assays provide cost- and labor-saving strategies to detect heteroduplexes. However, if the target genomic DNA fragment contains single nucleotide polymorphisms (SNPs) or allelic mutations, both PAGE and qPCR-based assays may give rise to false positive results [8]. Especially, loxP site is only 34 bp long and the product of genome editing is tough to trace by these two protocols. In addition, PAGE and qPCR-based assays have higher requirements for equipment and are also not cheap enough for low-budget laboratories, especially when required for high-throughput screening methods. Although a cut-price method using tri-primer-based typing protocol was applied in some recent studies, it needs to carry out multi-step PCR and is easy to be misjudged when the product size of wild type (wt) and homozygote (flox/flox) is close or when the sample has been contaminated [9, 10].

In this study, we developed a novel and rapid one-step genotyping method based on tetra primer-paired PCR amplification. The primer set is composed of a pair of common primers, one wt-specific primer and one loxP-specific primer. The PCR products for wt, heterozygote (wt/flox), and flox/flox alleles are clearly distinguished by agarose gel electrophoresis, which demonstrates a satisfactory advantage over the methods reported previously.

## Materials and Methods

### Animals

**Fig. 1:**
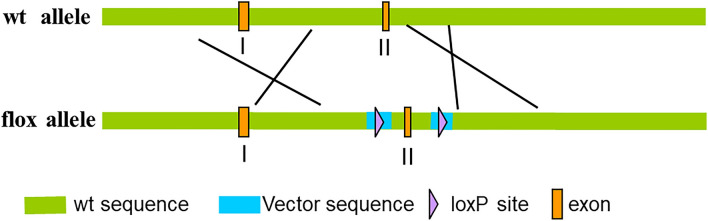
The genomic loci of loxP allele, and a targeting strategy of the Cre-loxP system

By cooperating with GemPharmatech Co., Ltd. (Nanjing, China), we successfully generated *Mbd2*^flox/flox^, *Pdia3*^flox/flox^ and *Wtap*^flox/flox^ mice via CRISPR/Cas9 tool. The targeting strategy is shown in Fig. 1. Meanwhile, two of those transgenic mice, *Mbd2*^flox/flox^ and *Pdia3*^flox/flox^, have been utilized and reported in our previous studies [11, 12].

For *Mbd2*^flox/flox^, given the gene has multiple transcripts, the shared exon2 (160 bp) was selected as the flox region. Then target sites were designed on the intron regions flanking exon2, and a targeting vector was constructed at the same time. Two pairs of loxp sites were integrated into the upstream and downstream of exon2 through homologous recombination. The small guide RNA (sgRNA) sequences are as follows: Mbd2-sgRNA-L-1: GAA TGC ACA TAG TCT GGT ATA GG; Mbd2-sgRNA-L-2: CGT TTG GAA TGC ACA TAG TCT GG; Mbd2-sgRNA-R-1: ACC CCA GCG CGC CGC CCT TGT GG; Mbd2-sgRNA-R-2: TCT CCA CAA GGG CGG CGC GCT GG.

For *Pdia3*^flox/flox^, given the gene has only one transcript, the exon2 (79 bp) was selected as the flox region. The sgRNA sequences are as follows: Pdia3-sgRNA-1: CTA CAG TCA GTG CAA TAG AGG; Pdia3-sgRNA-2: TTG AGA CAC CAA GTA AGT ACT GG.

For *Wtap*^flox/flox^, given the gene has multiple transcripts, the shared exon3 (89 bp) was selected as the flox region. The sgRNA sequences are as follows: Wtap-sgRNA-1: AAG GAC ACT TGG ACG GCT CAA GG; Wtap-sgRNA-1: CCA CCA AGG GAT AGG AAT ACT GG.

For in vitro fertilization, a total 2 male and 15 female C57BL/6 J mice were used to obtain oosperms. After mixing the 10 ng/µl sgRNA, 20 ng/µl Cas9 mRNA and 50 ng/µl donor DNA, they were co-injected into the cytoplasm of the in vitro fertilized oosperms via microinjection. After culturing for 1–2 h in vitro, the surviving oosperms were transplanted into the fallopian tubes of pseudo pregnant female mice. Then, the two mothers were housed in each cage under controlled temperature (22–26 °C) and humidity (50–65%) with food pellets and water freely available.

All mice were housed in the SPF facility at Tongji Hospital with the temperature controlled at 22–26 ℃ and humidity at 50–65%. All studies were conducted under the NIH guidelines and approved by the Animal Care and Use Committee (ACUC) in Tongji Hospital.

### DNA Preparation and PCR Amplification

The genomic DNA (gDNA) was prepared from the toe clipping of 2-week-old pups. To extract DNA, the alkaline lysis method was performed according to the previous report [13]. Briefly, the toe clipping of mouse was treated with 100 µl lysis solution (25 mM NaOH and 0.2 mM EDTA) and incubated for 45 min at 95 ℃. Following digestion, 100 µl 40 mM Tris–HCl pH 8.0 was added. After mixing the samples by brief vortex, 5 µl of the supernatant was used for PCR in a 15 µl reaction system.

For PCR, mixing the prepared gDNA samples with Taq Plus master Mix polymerase (Vazyme, P212-01, Nanjing, China), and 1 µl of each of the customized primers were added per tube. Then, PCR reactions for genotyping were carried out under the following conditions: 94 °C for 3 min, 35 cycles at 94 °C each for 30 s and annealing temperature (56 °C for *Mbd2* and *Wtap*, and 58 °C for *Pdia3*) for 40 s, followed by extension at 72 °C for 30 s and final extension at 72 °C for 10 min.

After PCR amplification, the gene products were subjected to 2% Tris Borate EDTA (TBE) agarose gels stained with GoldView (TsingKe Biotech, Beijing, China). After running under 180 V voltage condition, the amplicons were visualized on agarose gels using an UV illuminator (BioRad, Hercules, CA, USA). Hundred bp DNA ladder (Vazyme, Nanjing, China) was used as the size standard. The PCR primer synthesis in this study was performed by TsingKe Biotech Co. Ltd. (Beijing, China). Full-length gel images are presented in Supplementary Figs. 1–3.

### DNA Sequencing Analysis

The genotypes of the transgenic mice were further validated by Sanger sequencing with the same forward primers used in the PCR reaction at the TsingKe Biotech Co. Ltd. (Beijing, China). The obtained sequences were subjected to the NCBI BLAST (http://blast.ncbi.nlm.nih.gov/) for homology analysis.

### Statistical Analysis

No statistical method was used in this study.

## Results

### Strategy for Tetra Primer-Paired PCR Amplification System

**Fig. 2:**
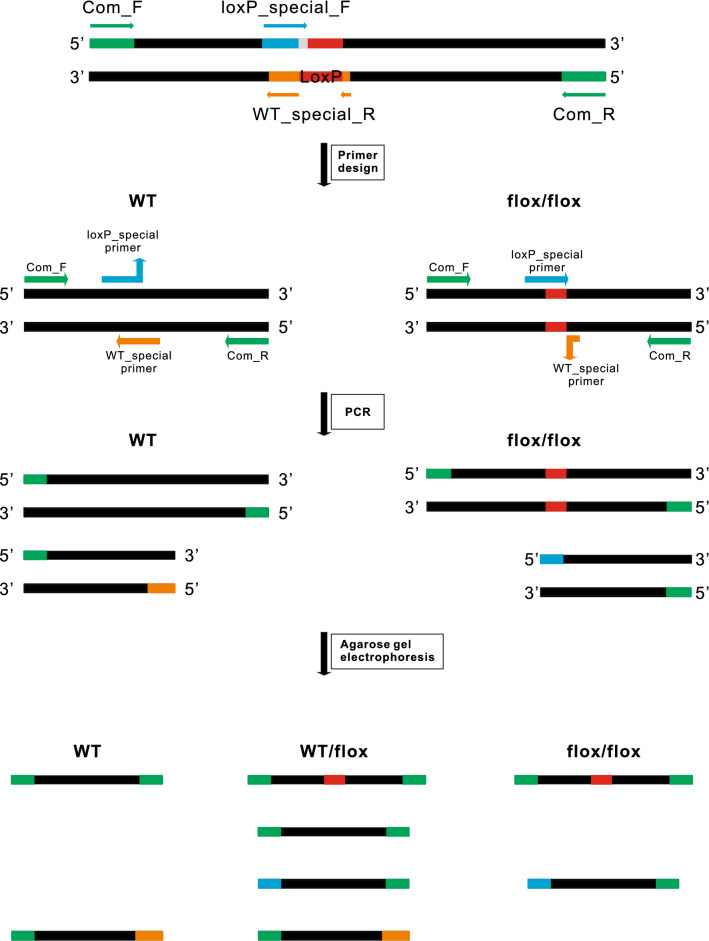
Schematic representation of one-step genotyping of the tetra-primer PCR system. The tetra primers are composed of a pair of common primers, a wt-specific primer and a loxP-specific primer. The green block is the common primer amplified sequence; the blue block is the wt-specific primer amplified sequence; the orange block is the loxP-specific primer amplified sequence; the red block is the loxP site

Tetra primer-paired PCR approach is applied as shown in Table 1 and Fig. 2. The tetra primers contain a pair of common primers, which target the sequence outside the editing site, one wt-specific primer which targets the wt allele sequence with at least 2 bp specifically matched to its 3′ end, and one loxP-specific primer which targets the loxP element with at least 2 bp specifically matched to its 3′ end. Wt-specific and loxP-specific primers are designed in reverse directions, they would pair with one common primer separately, amplify a PCR product with lower molecular size. The two specific bands have different sizes of at least 80 bp to make them distinguishable on agarose gels.

**Table 1:** Sequences of primers used for polymerase chain reaction

Gene	Primer sequence (5'‑3')	Tm	GC%
Mbd2_Com_F	CCTGCTCGTTGACAGGGTTATC	61.2	54.5
Mbd2_Com_R	GGTCAACAGCATTTCCCAGGTA	61.5	50.0
Mbd2_wt_R	GCGTTTGGAATGCACATAGTCTG	62.3	47.8
Mbd2_loxP_F	GTTATAAGCCTATACCAGACATAACTTCGT	59.6	35.7
Pdia3_Com_F	GCTGGAGTCTATTCCCTGTGTGT	59.9	52.2
Pdia3_Com_R	GCTTACTGCTAAGCCTAAGGACTCA	61.2	48.0
Pdia3_loxP_F	GATATAGCATCCTACAGTCAGTGCAAAT	62.1	39.3
Pdia3_wt_R	TCACCCTTTTCCACCTCTATTGC	62.4	47.8
Wtap_Com_F	CACTTGGACGGCTCAAGGATG	62.7	57.1
Wtap_Com_R	AGGGATGGCATAATGGTAAGTAGG	61.0	45.8
Wtap_wt_R	TGGAACTCTTCAGAGAACCAGTTTAGT	61.7	40.7
Wtap_loxP_F	TCTGACTGATTAGGTATTGCGAACTATAA	62.2	34.5

After a single PCR reaction, no matter which genotype the DNA template belongs to, the two common primers will generate a PCR product with a higher molecular size that can be regarded as an internal control. When the DNA template comes from homogenous mice with a wt or loxP sequence, the wt-specific or loxP-specific primer would pair with one common primer and amplify a PCR product with lower molecular size. Moreover, the shorter bands amplified by the special primer have different sizes for wt and flox. The DNA template from heterozygotes is similar to a mixture of wt and mutation, so four bands can be amplified after PCR reaction, while only three bands are visible as the internal control bands between wt and loxP alleles have only ~ 34 bp difference.

### Phenotypic Confirmation of Genotyping

To test the feasibility of our system, three different kinds of transgenic mice constructed by the CRISPR-Cas9 tool were used, including *Pdia3*^flox/flox^, *Wtap*^flox/flox^, and *Mbd2*^flox/flox^.

**Fig. 3:**
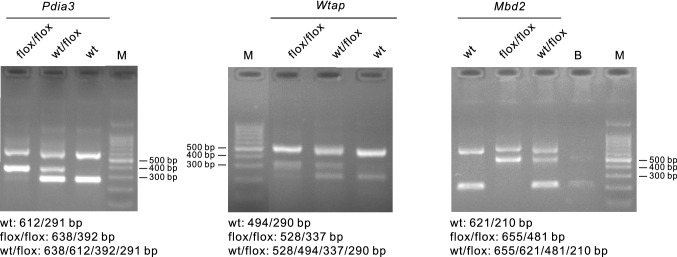
Genotyping of the three transgenic mice, *Pdia3*^flox/flox^, *Wtap*^flox/flox^ and *Mbd2*^flox/flox^. The PCR product including wild-type (wt), heterozygote (wt/flox) and homozygote (flox/flox), was analyzed on 2% Tris Borate EDTA (TBE) agarose gels stained with GoldView. flox, flanked by loxP; B, blank (water only); M, DNA marker

As shown in Fig. 3, for *Pdia3*^flox/flox^, the expected PCR product patterns were two bands (612/291 bp) for wt, four bands for wt/loxP (638/612/392/291 bp), and two bands for loxP/loxP (638/392 bp). For *Wtap*^flox/flox^, the expected PCR product patterns were two bands (494/240 bp) for wt, four bands (528/494/337/240 bp) for wt/loxP, and two bands (528/337 bp) for loxP/loxP. For *Mbd2*^flox/flox^, the expected PCR product patterns were two bands (621/210 bp) for wt, four bands (655/621/481/210 bp) for wt/loxP, and two bands (655/481 bp) for loxP/loxP.

### Confirmation of Genotyping Results by Sequencing

**Fig. 4:**
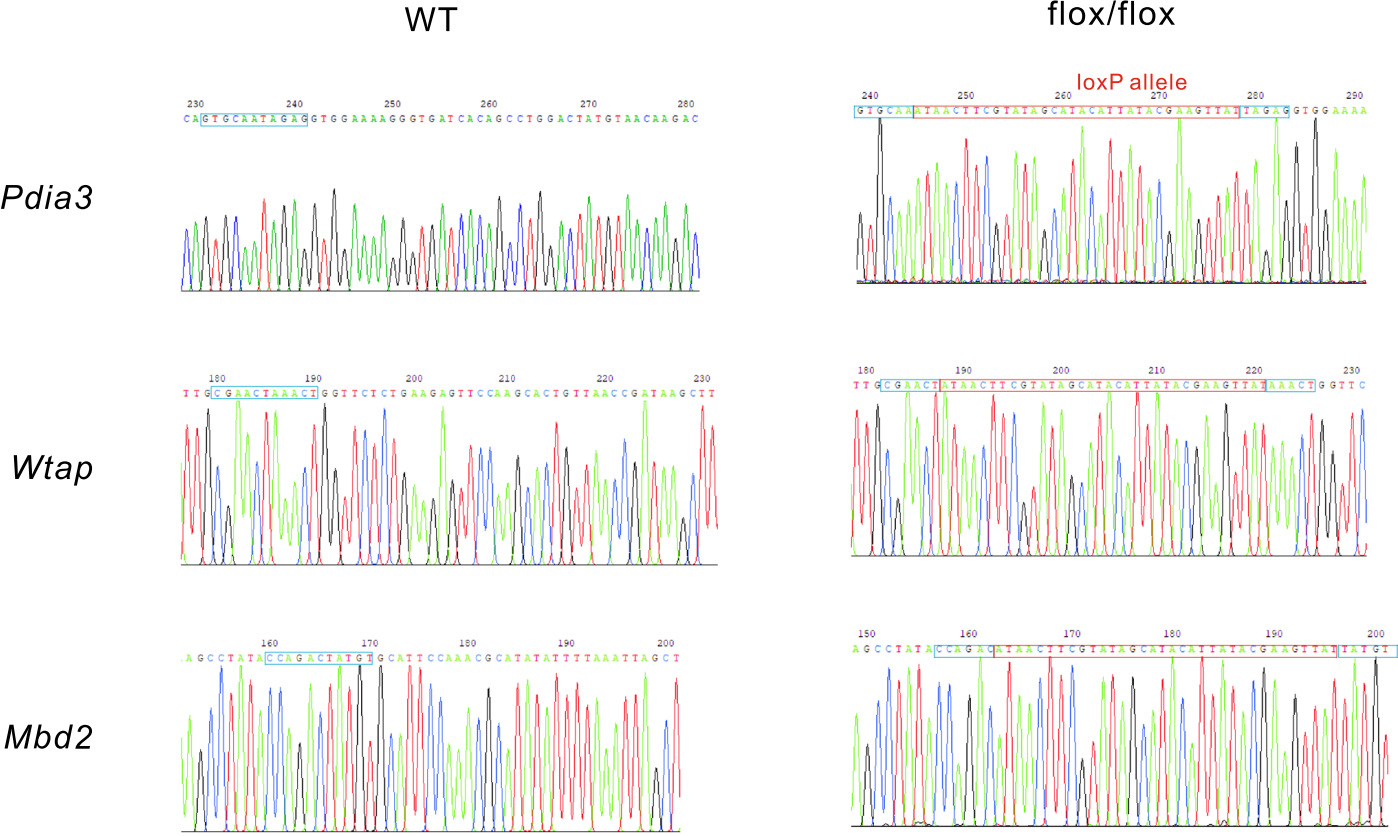
Confirmation of genotyping results by sequencing. The visible internal control band of the sample, including *Pdia3*^flox/flox^, *Wtap*^flox/flox^, and *Mbd2*^flox/flox^, has been cut and sent for sequencing. The red block is the loxP site and the blue block is the wild-type sequence

Since the genotyping results have been presented via agarose gel electrophoresis, we checked whether the loxP allele was correctly amplified by DNA sequencing. The visible internal control band of each sample was cut and sent for sequencing. Results showed that in all flox/flox bands, an intact and 34 bp long loxP element was detected, and this segment was undetectable in the wt group (Fig. 4).

## Discussion

With the development of CRISPR/Cas9 gene-editing tool, it has brought great convenience and efficiency to the establishment of transgenic mice [14]. However, for genotyping, the loxP site is too small and difficult to separate from the baseband when the resulting product was resolved by standard agarose gel electrophoresis.

Currently, several techniques have been developed and applied to aid in the genotyping of transgenic animals produced by CRISPR-Cas9, including Sanger sequencing of PCR products [3], T7 endonuclease I (T7E1) cleavage assay [15, 16], PAGE double-stranded detection [8], qPCR-based heterogeneous analysis and multi-step PCR-based typing protocol [5, 9, 10, 17, 18]. However, Sanger sequencing is time-consuming and expensive; T7E1 requires pre-testing of PCR products on a 1.5% agarose gel which is easy to mismatch and cause false-negative results. Likewise, both PAGE double-stranded detection and qPCR-based heterogeneous analysis are inefficient and labor-intensive. In addition, a recent study developed a slightly simpler method, but it requires two steps and the products need to be separated by 4–6% agarose gel [10].

In this study, we have developed a novel one-step PCR-based genotyping method using four special primers to rapidly identify wt, wt/flox, and flox/flox alleles. Our genotyping approach presents several advantages over currently used methods, including: (1) Simple and time-saving because of the one-step PCR amplification; (2) The results are more reliable when performing a large amount of genotyping, since the presence of the internal control can tell us the failure of PCR amplification. Meanwhile, the difference in the depth of the special band can tell us the minor contamination from other samples, especially, traces of aerosols can also be distinguished (e.g. as shown in Fig. 2S, there were minor contaminations in flox/flox samples). (3) Given the contamination can be distinguished, the requirements for the purity of DNA samples are not stringent, therefore, alkaline lysis method can be used for DNA extraction, which will save lots of costs; (4) The PCR reaction becomes economical since the mediocre Taq mix polymerase, instead of the high fidelity ones, can be used.

Noteworthy, to obtain the optimal genotyping result, there are several tips to be paid for attention: (1) Primers were designed such that all primers used in an assay had a difference of melting temperature (Tm) within 1 °C; (2) The size of the common products is better to set at a difference more than 80 bp, so that it can be easily distinguished on the 2% agarose gel; (3) This method is applicable to detect a wide array of small indel mutations of > 2 bp. In addition, the number of the expected products of DNA templates for heterozygote was four in reality. Due to the low sensitivity of agarose gel electrophoresis, only three bands can be clearly observed, but it does not affect the judgment of the result.

Taken together, our method which requires only one-step PCR amplification is more direct, simple and low-cost. Furthermore, we believe that our tetra primer strategy is not only confined to the *loxP* mice, but can also be applied to identify other kinds of indels.

## Limitations

Primer design is critical for setting up the PCR condition, the dimer of four primers and cross dimer between each two of them need to be avoided, and the maximum difference of annealing temperature of all four primers needs to be limited to 1 centigrade for a better amplification ratio. As the conditional knockout mice usually have two loxP alleles flanked with exons, there are four choices to design the wt-specific primer and loxP-specific primer, we can always find a suitable set of primes for the loxP-based conditional knockout mice generated by CRISPR-Cas9 technology.

## Supplementary Information

Below is the link to the electronic supplementary material.

Supplementary file1 (DOCX 755 KB)

## Data Availability

The authors state that all data necessary for confirming the conclusions presented in the article are represented fully within the article.

## References

[1] Sauer B, Henderson N (1988). Site-specific DNA recombination in mammalian cells by the Cre recombinase of bacteriophage P1. Proceedings of the National Academy of Sciences of the United States of America.

[2] Sander JD, Joung JK (2014). CRISPR-Cas systems for editing, regulating and targeting genomes. Nature Biotechnology.

[3] Zhao Y, Karan R, Altpeter F (2021). Error-free recombination in sugarcane mediated by only 30 nucleotides of homology and CRISPR/Cas9 induced DNA breaks or Cre-recombinase. Biotechnology Journal.

[4] Mashal RD, Koontz J, Sklar J (1995). Detection of mutations by cleavage of DNA heteroduplexes with bacteriophage resolvases. Nature Genetics.

[5] Thomsen N, Ali RG, Ahmed JN, Arkell RM (2012). High resolution melt analysis (HRMA); A viable alternative to agarose gel electrophoresis for mouse genotyping. PLoS ONE.

[6] Vouillot L, Thelie A, Pollet N (2015). Comparison of T7E1 and surveyor mismatch cleavage assays to detect mutations triggered by engineered nucleases. Genes Genomes Genetics.

[7] Shen B, Zhang W, Zhang J, Zhou J, Wang J, Chen L, Wang L, Hodgkins A, Iyer V, Huang X, Skarnes WC (2014). Efficient genome modification by CRISPR-Cas9 nickase with minimal off-target effects. Nature Methods.

[8] Zhu X, Xu Y, Yu S, Lu L, Ding M, Cheng J, Song G, Gao X, Yao L, Fan D, Meng S, Zhang X, Hu S, Tian Y (2014). An efficient genotyping method for genome-modified animals and human cells generated with CRISPR/Cas9 system. Science and Reports.

[9] Zarlenga DS, Chute MB, Martin A, Kapel CM (2001). A single, multiplex PCR for differentiating all species of Trichinella. Parasite.

[10] Bhattacharya D, Van Meir EG (2019). A simple genotyping method to detect small CRISPR-Cas9 induced indels by agarose gel electrophoresis. Science and Reports.

[11] Li N, Luo X, Yu Q, Yang P, Chen Z, Wang X, Jiang J, Xu J, Gong Q, Eizirik DL, Zhou Z, Zhao J, Xiong F, Zhang S, Wang CY (2020). SUMOylation of Pdia3 exacerbates proinsulin misfolding and ER stress in pancreatic beta cells. Journal of Molecular Medicine (Berlin, Germany).

[12] Wang Y, Zhang L, Wu GR, Zhou Q, Yue H, Rao LZ, Yuan T, Mo B, Wang FX, Chen LM, Sun F, Song J, Xiong F, Zhang S, Yu Q, Yang P, Xu Y, Zhao J, Zhang H, Xiong W, Wang CY (2021). MBD2 serves as a viable target against pulmonary fibrosis by inhibiting macrophage M2 program. Science Advances.

[13] Canene-Adams K (2013). General PCR. Methods in Enzymology.

[14] Mianne J, Codner GF, Caulder A, Fell R, Hutchison M, King R, Stewart ME, Wells S, Teboul L (2017). Analysing the outcome of CRISPR-aided genome editing in embryos: Screening, genotyping and quality control. Methods.

[15] Cho B, Kim SJ, Lee EJ, Ahn SM, Lee JS, Ji DY, Lee K, Kang JT (2018). Generation of insulin-deficient piglets by disrupting INS gene using CRISPR/Cas9 system. Transgenic Research.

[16] Nanjidsuren T, Park CW, Sim BW, Kim SU, Chang KT, Kang MH, Min KS (2016). GRK5-knockout mice generated by TALEN-mediated gene targeting. Animal Biotechnology.

[17] Chen S, Gong P, Zhang J, Shan Y, Han X, Zhang L (2021). Use of qPCR for the analysis of population heterogeneity and dynamics during Lactobacillus delbrueckii spp. bulgaricus batch fculture. Artificial Cells, Nanomedicine, and Biotechnology.

[18] Creane M, Howard L, O'Brien T, Coleman CM (2017). Biodistribution and retention of locally administered human mesenchymal stromal cells: Quantitative polymerase chain reaction-based detection of human DNA in murine organs. Cytotherapy.

