# Supplementary material for: One-Step Genotyping Method in loxP-Based Conditional Knockout Mice Generated by CRISPR-Cas9 Technology
Source: Mol Biotechnol. 2022 May 3;64(11):1227–33. doi: 10.1007/s12033-022-00500-5 (PMC9515137; doi:10.1007/s12033-022-00500-5)
Supplement: Supplementary file 1 — Supplementary file1 (DOCX 755 KB) [file 12033_2022_500_MOESM1_ESM.docx]

**One-step Genotyping Method in loxP Based Conditional Knockout Mice Generated by** **CRISPR-Cas9 Technology**

He Zhu ^1, #^, Siqian Liu ^1, #^, Wenxi He ^2^, Fei Sun ^1^, Yang Li ^1^, Ping Yang ^1^, Qilin Yu ^1^, Shu Zhang ^1,*^ Cong-yi Wang ^1,*^

^1^ The Center for Biomedical Research, NHC Key Laboratory of Respiratory Diseases, Department of Respiratory and Critical Care Medicine, Tongji Hospital, Tongji Medical College, Huazhong University of Science and Technology, Wuhan, Hubei Province, China.

^2^ Department of Pharmacy, Tongji Hospital, Tongji Medical College, Huazhong University of Science and Technology, Wuhan, Hubei Province, China.


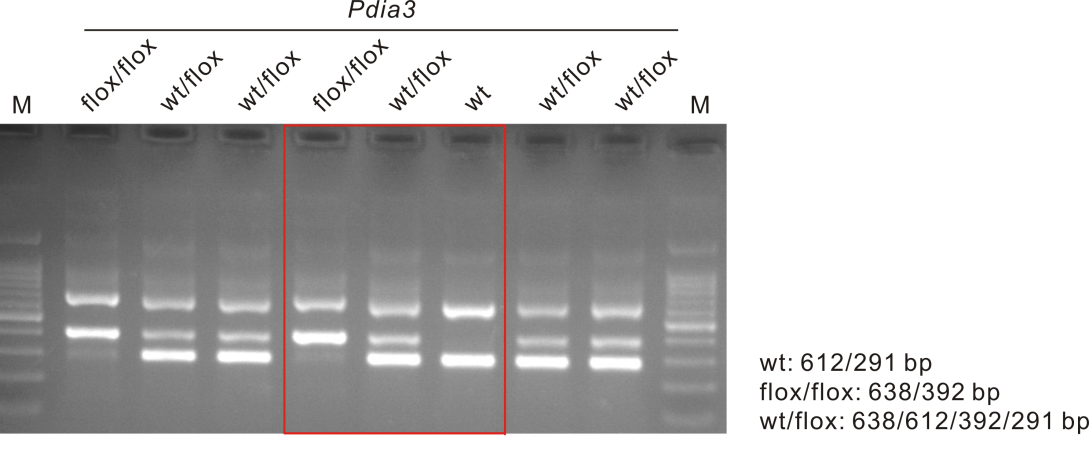


**Figure 1S** Original images of gels shown in this study. The red box is the interception area of Figure 3A. M, DNA marker.


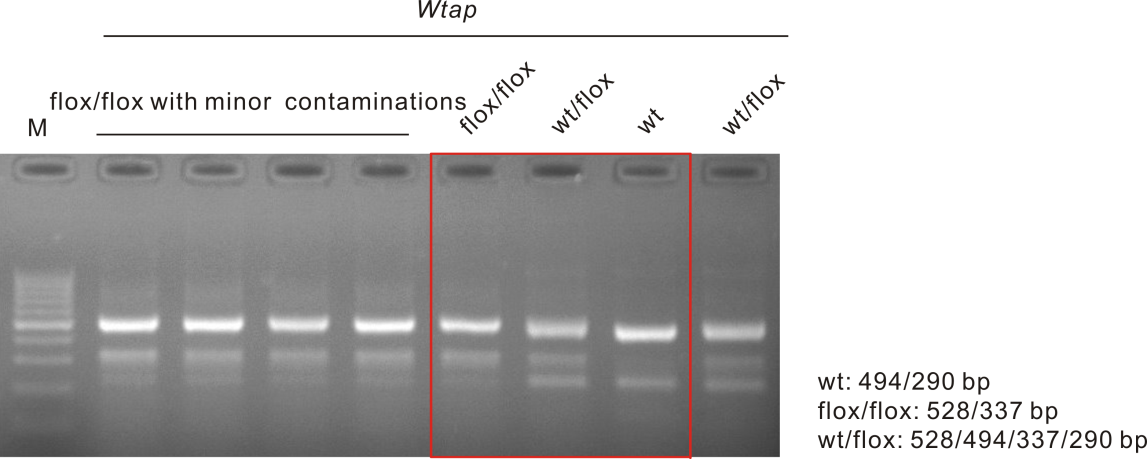


**Figure 2S** Original images of gels shown in this study. The red box is the interception area of Figure 3B. We can found that there were minor contaminations in the first four samples. But it did not affect our judgment that they were flox/flox samples, because the loxP-specific band (337 bp) was deeper than wt-specific band (290 bp). If the loxP-specific band is as deep as wt-specific band, the samples are wt/flow. M, DNA marker.


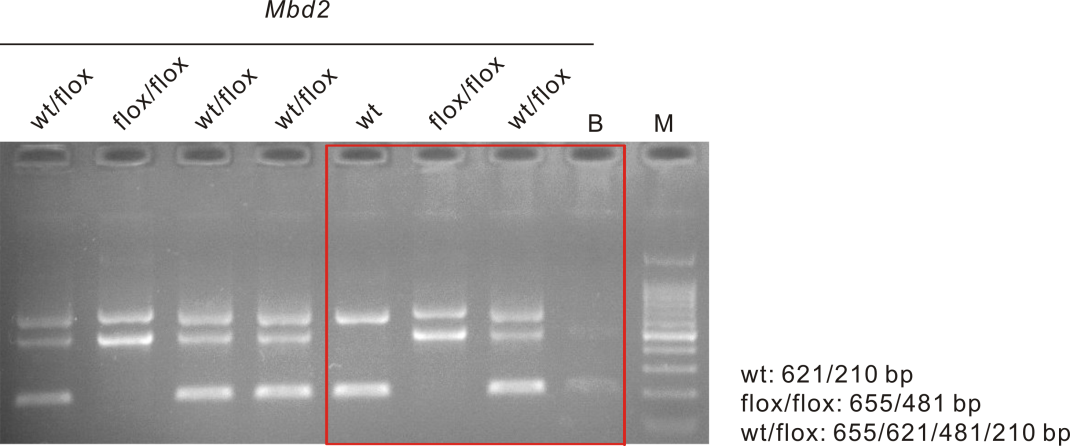


**Figure 3S** Original images of gels shown in this study. The red box is the interception area of Figure 3C. B, blank (water only); M, DNA marker.
